# Painful and non‐painful diabetic polyneuropathy: Clinical characteristics and diagnostic issues

**DOI:** 10.1111/jdi.13105

**Published:** 2019-07-29

**Authors:** Sandra Sif Gylfadottir, Danita Weeracharoenkul, Signe Toft Andersen, Supranee Niruthisard, Sompongse Suwanwalaikorn, Troels Staehelin Jensen

**Affiliations:** ^1^ Danish Pain Research Center Aarhus University Aarhus Denmark; ^2^ Pain Management Research Unit Department of Anesthesiology Faculty of Medicine King Chulalongkorn Memorial Hospital Chulalongkorn University Bangkok Thailand; ^3^ Department of Public Health Aarhus University Aarhus Denmark; ^4^ Department of Medicine Faculty of Medicine King Chulalongkorn Memorial Hospital Chulalongkorn University Bangkok Thailand; ^5^ Department of Neurology Aarhus University Hospital Aarhus Denmark

**Keywords:** Clinical characteristics, Diabetic neuropathy, Diagnosis

## Abstract

Diabetic neuropathy (DN) is a common complication of diabetes and can be either painful or non‐painful. It is challenging to diagnose this complication, as no biomarker or clear consensus on the clinical definition of either painful or non‐painful DN exists. Hence, a hierarchical classification has been developed categorizing the probability of the diagnosis into: possible, probable or definite, based on the clinical presentation of symptoms and signs. Pain is a warning signal of tissue damage, and non‐painful DN therefore represents a clinical and diagnostic challenge because it often goes unnoticed until irreversible nerve damage has occurred. Simple clinical tests seem to be the best for evaluation of DN in the general care for diabetes. Screening programs at regular intervals might be the most optimal strategy for early detection and interventions to possibly prevent further neuronal damage and to lower the economic burden of this complication.

## Introduction

Diabetes and its complications represent major and increasing challenges to healthcare systems worldwide. According to the International Diabetes Federation, 425 million people worldwide aged ≥20 years had diabetes in 2017, and this number is expected to increase to 629 million by 2045^1^. This development also applies to developing countries; for example, Asia, where there has been a dramatic increase in diabetes prevalence2. According to the International Diabetes Federation, it is estimated that 82 million adults have diabetes in South East Asia^1^.

Diabetic neuropathy (DN) represents a common, disabling and, until recently, largely neglected problem affecting approximately 50% of patients with diabetes at some point^3^, ^4^, ^5^, ^6^. A major problem with DN is that once it has developed and been complicated by, for example, ulcers and Charcot foot, it is difficult to reverse, and patients face an increased risk of amputations associated with increased mortality^7^, ^8^, ^9^, ^10^. It is therefore essential to detect symptoms or signs of DN as early as possible to implement interventions with a possibility of avoiding further neuronal damage. This has been stressed in several previous reviews^4^, ^5^, ^7^, ^8^, ^10^, ^11^. However, the detection of DN is challenging in clinical practice due to the lack of a clear consensus for the definition and optimal clinical assessments to diagnose DN^6^, ^12^, ^13^. Similar differences have been encountered for pain, where the criteria for neuropathic pain have also varied considerably^14^, ^15^.

The present review addresses the clinical characteristics of painful and non‐painful DN, and the possibilities for diagnosing these conditions. As the majority of patients with type 2 diabetes are treated in general practice, we focused on proposals for simple assessments of DN outside specialized hospital clinics.

## Definitions and classification of diabetic neuropathy

**Figure 1:**
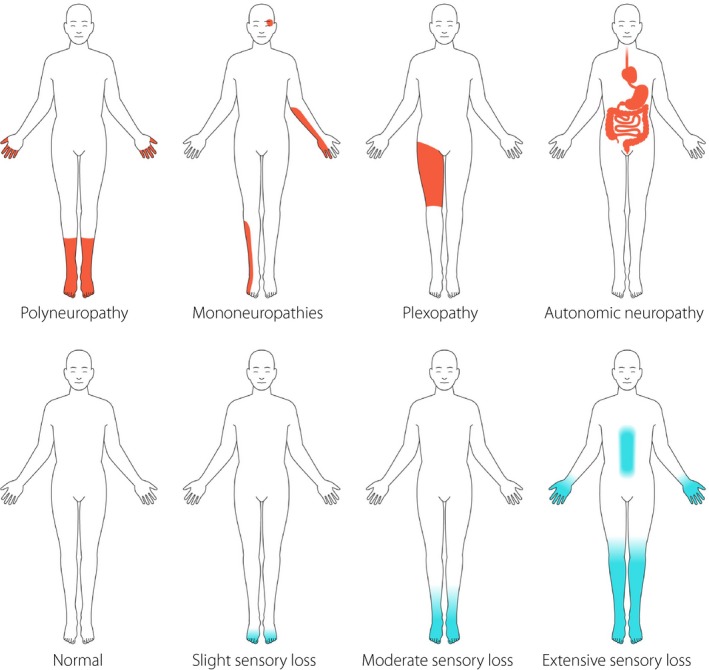
Clinical presentation of most common variants of diabetic neuropathy (upper panel) and the gradual progression of sensory changes (lower panel) in the most common form of diabetic neuropathy: diabetic polyneuropathy.

The phenotype of DN is heterogeneous. The most common form of DN is a chronic symmetrical length‐dependent sensorimotor polyneuropathy, termed diabetic polyneuropathy (DPN), which accounts for 75–90% of all DN cases. Other types of DN include autonomic neuropathy, diabetic radiculoplexopathy (formerly called diabetic amyotrophy), mononeuropathies and treatment‐induced neuropathies (Figure 1;Table 1)^4^, ^12^, ^16^. In the following, we will focus on DPN, which can be either painful (PDPN) or non‐painful. DPN is among other factors attributable to hyperglycemia, hyperglycemia‐associated metabolic derangement, dyslipidemia and microvessel alterations^17^. A broad and simple definition of DPN is “the presence of symptoms and/or signs of peripheral nerve dysfunction in people with diabetes after the exclusion of other causes”^6^. According to the International Association for the Study of Pain, neuropathic pain is pain caused by a lesion or disease of the somatosensory system^14^. Along these lines, PDPN can be defined as “pain caused by a lesion of the somatosensory system attributable to diabetes”. The minimal criteria for DN – whether painful or non‐painful – have, however, been a matter of debate for decades. The inconsistency of the definition used for DPN is reflected by varying prevalence estimates of this condition^10^, ^12^, ^18^, ^19^, ^20^.

**Table 1:** Classification of diabetic neuropathies

Diffuse neuropathy	Mononeuropathy	Radiculopathy	Other neuropathies
DPN primarily small fiber	Isolated cranial or peripheral neuropathy	Thoracic radiculoneuropathy	Pressure neuropathies
DPN primarily large fiber	Mononeuritis multiplex	Radiculoplexus neuropathy	CIDP
DPN mixed small and large fiber			Acute treatment induced neuropathy
DPN and autonomic neuropathy			

CIDP, chronic inflammatory demyelinating polyneuropathy; DPN, diabetic polyneuropathy.

## Clinical characteristics of non‐painful DPN

The development of DPN is insidious, usually starting in the toes and under the feet, and gradually ascending up the leg. When the symptoms have reached knee level, they usually start to occur in the fingertips, progressing further up in the hands and arms, reflecting the “dying‐back” progression of neuronal damage. Patients eventually present with a characteristic “stocking–glove” like distribution of neuronal dysfunction (Figure 1)^4^, ^21^, ^22^, ^23^. Consistent with the length‐dependent character of DPN, the distal nerve endings and intercostal nerves might even be affected, giving rise to a “daggert type” of sensory pattern. The temporal course of nerve fiber damage of different nerve fiber types in DPN is not clear. It is claimed that the most common early symptoms in DPN reflect early involvement of small fibers. Subsequently, the neuronal damage is proposed to progress to include large‐fiber dysfunction^6^. Small fiber involvement is usually painful, but might also give rise to negative symptoms, with selective loss of temperature and pain sensation. Large‐fiber dysfunction is characterized by numbness, “walking on wool” or a feeling as if the foot is “wrapped in paper”. With large‐fiber dysfunction, the gait might be insecure, either wide‐based or high stepping, and hold an increased risk of falls. In DPN, small‐ and large‐fiber dysfunction most commonly coexist, with combinations of large‐ and small‐fiber symptoms at clinical presentation, such as numbness, painful sensations, gait abnormalities and postural instability. However, as long prospective studies are scarce, no clear evidence exists for the proposed temporal course of neuronal dysfunction. We recently carried out a prospective study assessing this proposed temporal involvement of nerve fiber types in DPN, but found no evidence for this proposed hypothesis (Laura Linnea Määttä, personal communication).

In DPN, the characteristics of symptoms and signs are broadly divided into so‐called “negative” and “positive” symptoms^20^, ^24^, ^25^.

Negative symptoms are often described as numbness or a feeling of reduced sensation when walking: “It is like walking on cotton or foam”. Symptoms start slowly, and the unnoticeable loss of sensory function often remains unrecognized until irreversible nerve damage has occurred^25^, ^26^.

Positive symptoms range from non‐painful to painful symptoms^25^. Paresthesia is nonpainful sensations that are often described as tingling, prickling or ant‐like sensations. At other times it might feel like a pressing feeling, as if the foot is “squeezed into an unfitting shoe”, and the patient might have difficulty distinguishing between nonpainful and painful sensations. In most cases, symptoms are confined to the skin, but sometimes felt more deeply.

Signs of DPN include a reduced sensation to different sensory modalities involving small fibers (temperature and pinprick) and large fibers (vibration, position and cutaneous direction sense)^23^. The sensory loss overlaps partly or completely with symptoms; that is, the localization of sensory abnormalities also has a “stocking glove”‐ like distribution.

## Clinical characteristics of painful DPN

Pain represents a particular problem in PDPN, because it is associated with reduced quality of life and might compromise rehabilitation^27^, ^28^. Pain in PDPN can be neuropathic or non‐neuropathic (i.e., related or unrelated to the neuropathy). The non‐neuropathic types of pain include various types of musculoskeletal pain outside the foot and leg area. Non‐neuropathic foot pain might coexist with DPN and can be caused by, for example, peripheral artery disease, arthritis, spinal stenosis, local foot problems and other neuropathies. It can be difficult to distinguish non‐neuropathic pain from pain due to neuropathy per se. Neuropathic pain, whether caused by DPN, spinal cord injury or stroke, is located within the territory of the sensory abnormality, and might occupy the entire area of sensory abnormality or only a fraction of it^14^, ^29^, ^30^. This clinical pattern is different from those pains that are not associated with a specific nerve injury, where the pain distribution does not correspond to the innervation territory of a specific nerve, nerve root, group of fascicles or a segmental dermatome^14^, ^21^, ^24^. Neuropathic pains are generally of two types: spontaneous and evoked pain^21^, ^23^, ^24^. Spontaneous pains come in different forms: they can be shooting, shock‐like, aching, cramping, crushing, smarting or burning, and the pain can be present constantly or intermittently. In DPN, patients might describe their pain as unpleasant pricking or sticking sensations in the feet and toes. Evoked types of pain include allodynia (painful sensations elicited by non‐painful stimuli) and hyperalgesia (increased pain response by otherwise painful stimuli), which are both common in certain types of neuropathic pain, but considered to be relatively rare in DPN.

## Differences and similarities between PDPN and DPN

There has been an emerging interest in the potential differences in risk factors of PDPN and DPN, and the underlying pathogenesis of these conditions. Risk factors of both PDPN and DPN have recently been elegantly reviewed by Spallone and Greco^31^. They stated female sex, smoking, older age, overweight and a longer duration of diabetes as risk factors for both PDPN and DPN^31^. However, findings regarding older age and female sex as risk factors for PDPN and DPN are inconsistent in other studies^32^. As pointed out by Spallone and Greco^31^, previous studies have been heterogeneous in their design, study populations and applied definitions of DPN, rendering it difficult to compare risk factors across studies. Also, inconsistent findings for an association between pain and neuropathy severity exists^31^. Recently, three large cross‐sectional studies – a British^33^, a German–Czech^34^ and an Italian study^13^ have been carried out using comprehensive clinical evaluations of DPN including quantitative sensory testing (QST), skin biopsies (small nerve fiber quantitation), nerve conduction studies and a general neurological evaluation. The definitions of DPN and PDPN as well as the study design were fairly similar in the three studies, and the population consisted mostly of patients with type 2 diabetes. They identified only few and inconsistent differences in risk factors between PDPN and non‐painful DPN.

The British Pain In Neuropathy Study (PiNS) aimed to identify sensory phenotypes of patients with PDPN and DPN^33^. The study included 191 patients, and DPN was defined as symptoms or signs of DPN together with abnormal nerve conduction velocity or abnormal intra‐epidermal nerve fiber density. PDPN was defined according to the International Association for the Study of Pain definition of neuropathic pain^15^, ^26^. That study showed no difference between PDPN and DPN regarding sex, age, body mass index and waist‐to‐hip circumference. Patients with moderate‐to‐severe PDPN had higher HbA1c levels and were younger compared to those with mild PDPN or DPN. In addition, they found pain being correlated with more severe DPN^33^. In contrast, the Italian multicenter study of 816 type 1 and type 2 diabetes patients found female sex to be a risk factor for PDPN using the same definition of PDPN and DPN as the PiNS study^13^. They showed that patients with type 2 diabetes had a higher risk of neuropathy compared with patients with type 1 diabetes. In addition, higher body mass index, longer diabetes duration, and higher HbA1c levels were risk factors for both PDPN and DPN^13^.

Raputova *et al*.^34^ studied 232 patients with DPN, and showed that female sex was a risk factor for PDPN and more severe DPN.

The PiNS study evaluated clinical characteristics of sensory dysfunction, and the relationship between signs and symptoms in PDPN and DPN using QST and structural neurological examinations. Patients with PDPN had more pronounced neuronal abnormalities compared with patients with DPN, and mainly sensory loss involving both small and large nerve fibers. A small fraction (15%) of patients with PDPN had brush‐evoked allodynia, not seen in patients with DPN. The sensory phenotype of socalled “irritable nociceptor” (i.e., preserved small‐fiber function and hyperalgesia) was rare^33^. Similar findings were seen in the study by Raputova *et al*.^34^

The strength of all three studies is the robust definition of PDPN and DPN, reflecting the highest level of certainty of the diagnosis including symptoms, signs and a confirmatory test^15^, ^26^. However, a number of limitations exist, as two of these studies are small, and all three studies included both type 1 and type 2 diabetes patients, which might have influenced the results, given that PDPN and DPN could have different underlying pathogenesis and phenotypes in type 1 and type 2 diabetes patients^35^. All three studies are cross‐sectional, and thus cannot determine the temporal relationship between risk factors and PDPN and DPN. Finally, the group comparisons in the studies were slightly different. The study by Raputova *et al*.^34^ and the PiNS^33^ study compared patients with DPN, mild PDPN and moderate‐to‐severe PDPN, whereas the Italian study compared patients without DPN and patients with DPN or PDPN^13^.

In summary, many studies have compared PDPN and DPN in order to identify risk factors for the development of pain, to show the mechanisms underlying pain with the ultimate goal of identifying interventions for PDPN. Only a few and not fully consistent risk factors have been identified for PDPN. The most consistent objective finding is that PDPN presents with more profound sensory loss than DPN.

Longitudinal studies of patients with diabetes could be useful to determine the temporal course of nerve fiber damage and related clinical characteristics of patients with PDPN and DPN.

## Diagnosis

Diabetic neuropathies are complex diseases, and no robust definition or gold standard exist that fully encompass the complexity and changing course of nerve fiber damage in DPN. The hierarchical classification of DPN by the Toronto criteria into possible, probable or definite addresses this issue^26^. By these criteria, possible *DPN* requires symptoms of decreased sensation (e.g., numbness or pricking feeling in the toes, feet or legs) or signs (i.e., symmetric decreased sensation or decreased or absent ankle reflexes). Probable DPN requires symptoms and signs, including two or more of the following: neuropathic symptoms, decreased distal sensation or decreased or absent ankle reflexes. Definite DPN requires abnormal nerve conduction studies or an abnormal validated measure of small fiber damage in combination with a symptom or a sign. Importantly, these criteria permit for the changing course of nerve fiber damage in DPN, but at the same time they also illustrate the clinical challenge that there is no specific measure to diagnose DPN in any individual at all times throughout the course of DPN.

**Figure 2:**
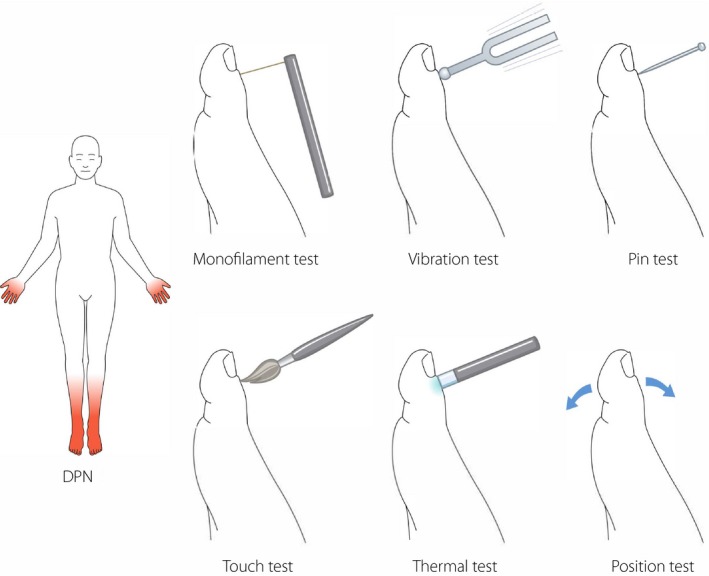
Diabetic polyneuropathy (DPN). Bedside tools for testing cutaneous sensation, both large fiber function: 10‐g monofilament, vibration with 128‐Hz tuning fork, touch and joint position, and small‐fiber function: cold and warm sensation, and pinprick.

The examination for DPN starts at the “bedside” with simple assessments of signs of neuropathy (Figure 2). In addition, the examination should include foot inspection, joint mobility testing and evaluation of motor function. Table 2 summarizes a list of symptoms and clinical signs and assessments proposed for the clinical evaluation of DPN.

**Table 2:** Characteristics of large and small fiber function and their assessment^23^, ^51^, ^53^, ^54^, ^55^

	Large fiber neuropathy	Small fiber neuropathy
Symptom	Numbness, tingling, gait instability	Burning pain, electrical shock, stabbing pain
Examination	Reflexes, proprioception, vibration	Temperature, pinprick sensation
Function	Pressure, balance, muscle strength	Pain sensation, protective sensation
Diagnostic test	Nerve conduction studies DPN Check^™^ (point‐of‐care device assessing sural nerve conduction) Neurothesiometer Vibrameter Tuning fork (128 Hz)	Quantitative sensory testing (QST) Intradermal nerve fiber structure Cornea confocal microscopy Laser Doppler imaging after noxious stimulus Sudomotor function Skin conductance measurement Microneurography

DPN, diabetic polyneuropathy.

### Clinical scoring systems for the screening of DPN

A number of scoring systems have been developed for the screening of DPN. The most widely used in previous clinical studies are as follow (Table 3).

**Table 3:** Scoring‐systems for diabetic polyneuropathy

Name of test (abbreviation)	Symptoms/signs	Items assessed	Reference
Toronto Clinical Neuropathy Score	Symptoms and signs	Pain, tingling numbness, reflexes, pin, touch, temperature, vibration, joint, muscle weakness, ataxia	36
Diabetic Neuropathy Symptom Score	Symptoms	Pain, numbness, tingling, ataxia	38
Neuropathy Symptom Score	Symptoms and signs	Muscle strength, sensory abnormality, autonomic symptoms	39
Neuropathic Disability Score	Signs	Vibration 12 Hz, temperature, pinprick, ankle reflex	22
Michigan Neuropathy Screening Instrument questionnaire	Symptoms	Pain, temperature, tingling, numbness and other questions	41
Michigan Neuropathy Screening Instrument examination	Signs	Foot inspection, ankle reflexes, vibration and light touch sensation	41
Utah Early Neuropathy Score	Signs	Muscle strength, pin, allodynia, ankle reflex, vibration, joint position	43
Michigan Diabetic Neuropathy Score	Symptoms and signs	MNSIQ + MNSI + nerve conduction studies	42
The neuropathy Impairment score of lower limbs	Symptoms and signs	Vibration, pinprick, touch, pressure, joint position, motor assessment, knee and ankle reflexes	42

MNSI, Michigan Neuropathy Screening Instrument; MNSIQ, Michigan Neuropathy Screening Instrument Questionnaire.

The Toronto Clinical Neuropathy Scoring System^19^, ^36^. This scoring system measures three parameters: neuropathic symptoms, knee‐ and ankle reflexes, and sensory testing applied to the dorsum of the first toe (light touch, pin prick, vibration, temperature and position sensation). A modification of the original scoring system came in 2009, where measurement of tendon reflexes was excluded from the score. The modified version was recently evaluated in a comparative study of seven neuropathy scoring systems in people with impaired glucose tolerance and controls, and showed the highest accuracy for detecting DPN when compared with a clinical examination fulfilling criteria of definite DPN (an area under the curve of 0.998)^37^. However, as that study assessed people with impaired glucose tolerance, the findings might not apply to cohorts of people with overt diabetes and DPN.

The Diabetic Neuropathy Symptom Score^38^ is a simple score, and other systems, including the Neuropathy Symptom Score with 17 different items^39^ and its extension Neuropathy Symptom Profile with even more items,^40^ exist.

The Michigan Neuropathy Screening Instrument^41^ consists of two parts: a patientadministered questionnaire and a clinical examination. The examination includes: foot inspection (deformities, dry skin, infection, fissures and ulcers), ankle reflex assessment, vibration sensation (128‐Hz tuning fork) and light touch sensation (10‐g monofilament) on the dorsum of the first toe^42^. This instrument was tested in a large cohort of 1,184 patients with type 1 diabetes and showed a high specificity (approximately 95%), but a lower sensitivity (approximately 43%) for a combined score including both parts of the instrument and compared with a clinical examination fulfilling the criteria for definite DPN^56^.

The Utah Early Neuropathy Scale (UENS)^43^ was designed as a simple and quick examination to identify early‐stage DPN. The UENS is based on assessments of the first toe extension vibration, proprioception and an extended examination of pinprick sensation including assessment in six different segments on the foot and lower limb. Allodynia and ankle reflexes are also assessed. The UENS puts high emphasis on loss of pinprick sensation, which accounts for 24 out of a maximum of 42 points for detecting DPN. The UENS was tested on 215 patients with or without DPN, and showed a higher sensitivity (92%) than the Michigan Diabetic Neuropathy Scale and the Neuropathy Impairment Score‐Lower Leg with a similar specificity as seen for these scores.

Because of the progressive nature of DPN, it is important to determine the severity of neuropathy, and follow up the course of the condition in patients. The Neuropathy Disability Score used in the North‐West Diabetes Foot Care Study^44^ classified neuropathy severity into three categories – mild, moderate and severe – based on objective semiqualitative measures (vibration, temperature, pinprick sensation and ankle reflexes). Positive signs, such as allodynia and hyperalgesia, are not scored and included in this score. Dyck *et al*.^45^ used another classification in which DPN is graded into four categories – 1a, 1b, 2a or 2b – based on symptoms, signs and nerve conduction studies. The limitation of this test is that it does not account for small fiber abnormalities. Dyck *et al*.^45^ also suggested an alternative approach to grading the severity of DPN by a composite score including clinical signs of DPN and nerve conduction studies or other neurophysiological measures.

### Pain

For PDPN, similar screening tools as the ones described for DPN have been developed. The Neuropathic Pain Questionnaire consists of 12 questions, and the German developed *Pain Detect* is based on a self‐administered questionnaire with nine different questions related to pain type^25^, ^46^. In addition, a body phantom is used to assess and quantitate the distribution of pain types. The *Leeds Assessment of Neuropathic Symptoms and Signs* (*LANS*) combines five questions with two examination items^47^. The French screening tool, *Douleur Neuropathique en 4 question* (*DN4*), includes seven questions and three examination items^25^, ^48^, and the Neuropathic Pain Symptom Inventory consists of 12 questions about neuropathic pain descriptors^49^.

The sensitivity of these screening tools is 80–85%, and the specificity a little higher. It has been pointed out that the screening tools fail to identify approximately 10–20% of patients with clinical neuropathic pain in general^50^. Although screening tools are useful for a quick and easy way to identify patients with neuropathic pain, a negative answer does not rule out PDPN.

### Clinical measures of PDPN and DPN

A number of other diagnostic tests are available for DPN, PDPN and neuropathic pain in general^51^, ^52^, ^53^, ^54^. These tests include skin biopsies with quantitation of intra‐epidermal and dermal nerve fibers^5^, measurements of small nerve fibers in the cornea using corneal confocal microscopy^56^, ^57^, and assessment of neurogenic flare with laser Doppler as a measure of small nerve fiber (C fiber) function^58^. In addition, assessment of sudomotor function and quantitative sensory tests exist^51^. Still, abnormal nerve conduction studies are considered the first objective and quantifiable measure of DPN. Nerve conduction studies usually include examination of distal latency, conduction velocity, and sometimes F‐wave latency of motor nerves (ulnar, peroneal and tibial) and sensory nerves (ulnar, radial and sural). There is no formal consensus for the definition of DPN by nerve conduction studies. However, it is generally accepted that abnormality of one or multiple parameters (values outside ±2.3 standard deviation) in one or several nerves is abnormal. Examinations should be evaluated against normative reference values taking age, skin temperature and patient height into account^45^. It is now recognized that nerve conduction studies have some limitations^59^. It requires the use of expensive devices not available in the general care of diabetes, the examination is timeconsuming and it cannot identify small nerve fiber damage. These limitations are recognized in the recent position statement from the American Diabetes Association in which it is stressed that the diagnosis of DPN is a clinical one only requiring nerve conduction studies in patients with clear motor deficit, an asymmetrical presentation of nerve fiber dysfunction or in patients suffering from falls^6^. The Toronto criteria for DPN do not include criteria for the definition of PDPN, but similar algorithms exists for diagnosing this condition^15^, ^60^.

## History of symptoms and clinical examination in primary and secondary care outside neurological units

From the perspective of primary care and secondary care outside neurological specialized units, a simple questionnaire for DPN is important. Three symptoms are considered highly suggestive of DPN: numbness, tingling and pain^61^. To assess the impact of symptoms, questionnaires should also include assessment of quality of life and sleep^62^. The agreement of symptoms and clinical abnormalities in DPN is relatively low, and thus symptom assessment should be combined with a clinical examination for DPN. If very limited time is available for consultation, a very brief and simple examination for DPN lasting only a few minutes has been developed^63^. Table 4 summarizes elements that should be included in the examination of DPN, including examination of skin, and musculoskeletal, vascular and neurological function. Skin examination should include inspection for ulcers, callosities and nail abnormalities. Musculoskeletal function assessment included evaluation of foot deformities of the forefoot (e.g., abnormal posture of toes and midfoot (incipient or frank Charcot deformities). The assessment of vascular function should include palpation of the dorsal pedal and the posterior tibial pulses, and capillary response (reduced if reddening does not occur within 2 s after release of finger pressure). The neurological examination should include assessment of large‐ and small fiber function. For large fiber function, the examination includes both motor‐ and sensory functions. For motor function, atrophy of small muscles in the feet, and strength of dorsal‐ and plantar flexion of the foot, toe and fingers are examined. Ankle and knee reflexes are recorded as normal, reduced or absent. Large sensory function includes assessment of vibration sense (128‐Hz tuning fork), position sense of the first toe and light touch perception (10‐g Semmes‐Weinstein monofilament). Assessment should be carried out on the dorsal part of the bony prominence of the first toe immediately proximal to the nail bed. The vibration sense is considered absent if the patient cannot feel the vibration. In some countries, a neurothesiometer is used to determine vibration sense. The device is applied to the dorsal aspect of the first toe and voltage is increase until a vibration is perceived. A value >25 V is considered abnormal^64^. The 10‐g monofilament test is carried out at the same site on the first toe, with the monofilament applied perpendicular to the skin and pressing until the filament buckles. The test is considered abnormal if the filament is not perceived. Note that the monofilament test is also used for identifying patients with a high risk of ulcer development. In these cases, the filament is applied to the plantar surface of the first, third and fifth metatarsal head, and to the plantar part of the distal first toe. Joint position is tested by dorsal and plantar flexing the first toe a few millimeters while the patient is closing their eyes. The failure to identify the correct position is considered abnormal. Small‐fiber function assessment should include pinprick and temperature sensation. For pinprick sensation a Neurotip^™^ or a sharp wooden pin is used. Failure to identify the prick sensation is abnormal. Lesioning of the skin should be avoided. For temperature sensation assessment, different devices, such as glass tubes filled with cold and warm water, thermorollers or a simple thermal tip device termed “Thermotip”, have been developed. Failure to sense a cool and warm sensation on the dorsum of the first toe or dorsum of the foot is considered abnormal. In case of distal abnormalities, the examination should identify the proximal level for involvement of sensory abnormalities.

**Table 4:** Examination of diabetic polyneuropathy

Skin examination	Musculoskeletal assessment	Vascular assessment	Neurological assessment
Callosity, cracking of skin	Hallux valgus	Foot pulses	Vibration sense (128‐Hz tuning fork)
Dry skin, sweating	Claw/hammer toes	Capillary response	Pinprick sensation (Neurotip^™^)
Infection in skin or nails	Charcot foot	Ankle‐brachial index	Temperature sensation (Thermotest, thermoroller, Tip Therm^®^)
Ulcers	Muscle wasting		Light touch sensation (10 g monofilament)
			Ankle and knee reflexes
			Muscle strength

Additional examination for allodynia is carried out by stroking with a brush or cotton wool to determine the presence of pain by non‐noxious dynamic stimulus. If pain is evoked, this indicates allodynia.

In the statement by The American Diabetes Association^6^, DPN is proposed to be diagnosed by a history of symptoms and assessment of either pinprick or temperature sensation (small‐fiber function) and vibration sensation using a 125‐Hz tuning fork (large‐fiber function).

## Conclusion

Diabetic neuropathy in type 2 diabetes presents in a painful and a non‐painful form. The non‐painful variant is the most dangerous because of its insidious nature, and gradual loss of sensation in the feet and lower limbs. Neuropathy might therefore go unnoticed by the patient until irreversible nerve damage has occurred, carrying an associated high risk for foot ulcers, foot deformities and limb amputation. Early detection of DN is therefore crucial, and simple screening assessments at regular intervals are suggested to be the best strategy in the general care for diabetes to possibly avoid further neuronal damage.

## Disclosure

T.S. Jensen has received consultancy fees from Pfizer, Biogen and Mundipharma. The other authors declare no conflict of interest.
